# Analysis of characteristics of unintentional injuries among hospitalized children and preventive strategies—based on adverse event data of a regional medical center in western China

**DOI:** 10.3389/fpubh.2025.1671279

**Published:** 2025-10-07

**Authors:** Tianlan Li, Jing Pu, Zhi Zeng, Dan Wen, Li Wan

**Affiliations:** Mianyang Central Hospital, School of Medicine, University of Electronic Science and Technology of China, Mianyang, Sichuan, China

**Keywords:** children hospitalized, unintentional injury, adverse events, preventive strategies, child

## Abstract

**Objective:**

To analyze the characteristics of unintentional injuries among hospitalized children in a regional medical center in western China and to put forward preventive strategies.

**Methods:**

The adverse event data of hospitalized children from January 1, 2015 to December 31, 2024 in our hospital were collected retrospectively. A descriptive statistical analysis was performed on the types, occurrence time and place, age of children, and injury severity of 428 unintentional injuries.

**Results:**

Among 428 cases of unintentional injuries, males predominated (63.3%). Children aged 1–3 years were most frequently affected (45.3%), followed by those under 1 year (34.8%). The most common injury types were treatment-related incidents (“Medical care”, 32.2%), falls from bed (25.9%), and mechanical forces (“EIMF”, 10.5%). Over half of injuries occurred during 12:00–24:00, with peaks at 18:00–24:00 (30.8%) and 12:00–18:00 (27.8%); most (90.2%) took place in wards. Age, time, and place were significantly associated with injury incidence (*p* < 0.05). No fatal (Level I) injuries occurred; most were mild (Level III), with severity significantly linked to injury sorts and place (*p* < 0.05).

**Limitations:**

This study has several limitations, future research should adopt prospective, multi-center designs to validate these findings and develop targeted interventions for different healthcare settings.

**Conclusion:**

Unintentional injuries among pediatric inpatients predominantly affect young children, especially males aged 1–3 years, and are closely linked to treatment procedures, environmental factors, and specific time periods. Most incidents are mild and occur in wards, highlighting the need for strengthened safety management in clinical settings, staff training during high-risk hours, and targeted parental education to mitigate preventable harm.

## 1 Introduction

Unintentional injuries in children, also known as accidental injuries, are defined as harm caused by external, sudden, and non-disease-related factors that are often unforeseen or underestimated (1). They represent a major public health challenge worldwide, posing a significant threat to children’s health, survival, and development (2). Although remarkable progress has been made in improving child survival—reflected by a global 60% decline in the under-five mortality rate (U5MR) over the past three decades—unintentional injuries remain a leading cause of preventable childhood deaths (3). Globally, it is estimated that approximately 2,000 families lose a child every day due to unintentional injuries (4). These figures highlight that despite the overall success in reducing child mortality, the burden of unintentional injuries among children persists as a pressing issue in global child health.

China, the world’s largest developing country, faces particularly severe challenges in preventing childhood unintentional injuries. Although the U5MR in China decreased dramatically from 54 per 1,000 live births in 1990 to 8 per 1,000 in 2018 (5), unintentional injuries have become the leading cause of death among children under 14 years, accounting for nearly 40–50% of all-cause child deaths (6). Each year, more than 50,000 Chinese children die due to such injuries, with the burden especially high in the 1–4-year age group (7). From 2016 to 2022, unintentional injuries accounted for 1.2% of deaths among children under five nationwide, with the burden highest in western China (3.5%), highlighting marked regional disparities (8). These trends emphasize the urgent need to strengthen prevention and control strategies, particularly in western regions.

Unintentional injuries among hospitalized children refer to accidental harms (such as falls, burns, traffic-related injuries, or drowning) that require hospital admission, with their causes, severity, and recovery outcomes often linked to factors like age, environmental safety, and family supervision, and being a key focus of pediatric injury prevention and clinical management. Extensive international research has explored the epidemiology, risk factors, and prevention of unintentional injuries among children, with studies focusing on race, fractures, falls, and burns (9, 10). In high-income countries, prevention programs and safety regulations have led to notable reductions in child injury-related deaths (11). In China, research has mainly concentrated on community- or household-based injury prevention, and some studies have examined overall epidemiological patterns (12, 13). However, few investigations have addressed hospital-based unintentional injuries among pediatric patients, despite their classification by the World Health Organization (WHO) as adverse events (AEs) associated with healthcare management (14). For this study, we define AE as an unintended injury to a patient resulting from medical intervention, healthcare actions, or inactions. Existing evidence suggests that up to one-third of hospitalized children may experience an AE, many of which are preventable (15). Yet, systematic data on the characteristics of unintentional injuries occurring within hospital settings in China remain limited.

Given these gaps, there is a clear need to better understand the epidemiology and characteristics of unintentional injuries in hospitalized children in China, particularly in regions with higher disease burdens such as western China. This study therefore aims to analyze the characteristics of adverse events associated with unintentional injuries in hospitalized children at a regional medical center in western China. By providing evidence-based insights, this research seeks to inform the development of targeted prevention strategies and to enhance pediatric patient safety management in the hospital setting.

## 2 Methods

### 2.1 Date sources

Data were obtained from the adverse event reporting system of a regional tertiary medical center in western China. The Department of Pediatrics at this hospital was established in 1958. Currently, it comprises five outpatient units and four inpatient wards. The outpatient services include specialized clinics, pediatric emergency care, pediatric fever clinic, day ward, and the Early Childhood Comprehensive Development Center (which includes child healthcare, developmental and behavioral pediatrics, assessment center, and rehabilitation intervention center). The inpatient wards consist of Pediatric Ward I, Pediatric Ward II, Pediatric Intensive Care Unit (PICU), and Neonatal Intensive Care Unit (NICU), covering a total of 14 pediatric subspecialties. The department has 209 open beds. The annual outpatient and emergency volume is approximately 300,000 visits, with around 12,000 inpatient discharges per year. Currently, there are 199 medical staff members in the department, including 58 physicians and 141 nursing personnel. We retrieved the hospitalization information of all patients with unintentional injuries through the Hospital Information System (HIS). We collected the demographic information of the patients, including age, sex, as well as the injury sort, time and place of occurrence, severity level of injury, and clinical outcomes.

### 2.2 Subjects

**Figure 1:**
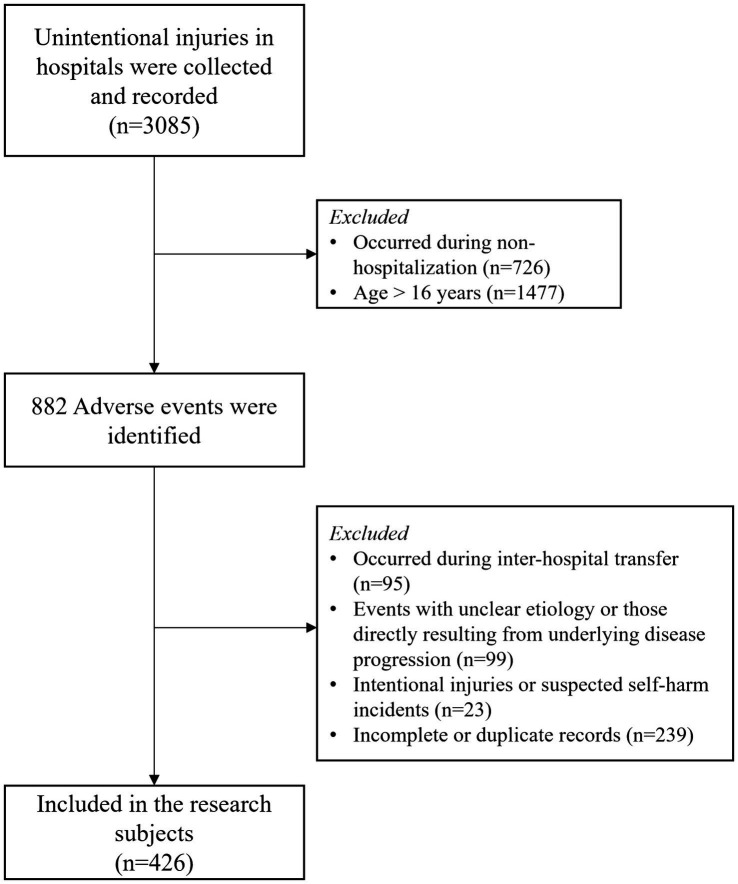
The screening process of the research subjects.

The study included unintentional injury events involving hospitalized children aged 0–16 years, reported between January 2015 and December 2024. Inclusion criteria comprised: Documented unintentional injury events occurring during hospital stay; complete records regarding injury sort, time, place, patient age, gender, and injury severity; age at the time of event ≤ 16 years. Exclusion criteria were: Injuries occurring during inter-hospital transfer; events with unclear etiology or those directly resulting from underlying disease progression; Intentional injuries or suspected self-harm incidents; Incomplete or duplicate records. The specific sample screening process is shown in Figure 1.

A structured screening process was implemented to identify eligible cases. Initially, all adverse event reports in the specified period were extracted. Two researchers independently reviewed each record according to the inclusion and exclusion criteria. Discrepancies were resolved through discussion or adjudication by a third reviewer. The final analytic sample consisted of 428 unintentional injury cases meeting all eligibility requirements.

### 2.3 Definition of unintentional injuries

Unintentional injuries were classified according to the World Health Organization’s International Classification of Diseases-10 (ICD-10). The codes used in this study are as follows: Fall involving bed (W06), Falls (W00-05, W07-19), Burn and scald (X00-X19), Exposure to inanimate mechanical forces (EIMF, W20-W49), Misadventures to patients during surgical and medical care (Medical care, Y60-Y69), Accidental deletion of drug or wrong drug given or taken in error (Drug, X40-49), Medical devices associated with adverse incidents in diagnostic and therapeutic use (Medical device, Y70-Y82). In particular, medical device (Y70-Y82) includes medical device breakdown or malfunctions that occur during procedure, after implantation, and ongoing use. Among them, FIM (W06), Falls (W00-05, W07-19), Burn and scald (X00-X19) and EIMF (W20-W49) all belong to the category of Other external causes of accidental injury (W00-X59). However, Medical care (Y60-Y69), Drug (X40-49) and Medical device (Y70-Y82) can be assigned or approximately assigned to Complications of medical and surgical care (Y40-Y84) class.

According to the commonly used injury severity classification standards in the world and the actual situation of hospitals, the severity of unintentional injury events was divided into Level I, II, III, and IV events (16, 17). Level I event: an unexpected death, or permanent loss of function due to natural processes other than disease; Level II event: impairment of patient’s body and function caused by nursing activities rather than the disease in the medical treatment process of the disease; Level III events: although erroneous facts occurred, no damage was caused to the body and function, or there were slight consequences that required no treatment for complete recovery; Level IV events: no erroneous facts were formed due to timely discovery.

### 2.4 Statistical analysis

Statistical analyses were conducted using SPSS software (version 22.0; IBM Corp., Armonk, NY, United States). Categorical variables are summarized as frequencies and percentages. Group comparisons for categorical data were performed using the chi-square (χ^2^) test. A two-sided *p*-value < 0.05 was considered statistically significant.

## 3 Results

### 3.1 Demographic data

The demographic characteristics of unintentional injuries among hospitalized children from 2015 to 2024 are summarized in Table 1. A total of 428 cases were included in the analysis. Gender distribution indicated a predominance of male patients (*n* = 271, 63.3%) compared to female patients (*n* = 157, 36.7%), resulting in a male-to-female ratio of 1.7:1. Across the years, males consistently accounted for a higher proportion of injury cases, with peaks observed in 2016 (19.6%) and 2015 (18.1%).

**Table 1:** Demographic characteristics of unintentional injuries among hospitalized children from 2015 to 2024.

Variables	2015	2016	2017	2018	2019	2020	2021	2022	2023	2024	Total
Gender											
Male	49 (18.1%)	53 (19.6%)	46 (17.0%)	30 (11.1%)	11 (4.1%)	17 (6.3%)	22 (8.1%)	20 (7.4%)	11 (4.1%)	12 (4.4%)	271 (100%)
Female	27 (17.2%)	25 (15.9%)	31 (19.7%)	19 (12.1%)	11 (7.0%)	13 (8.3%)	11 (7.0%)	6 (3.8%)	7 (4.5%)	7 (4.5%)	157 (100%)
Age											
0–1 year	29 (19.5%)	33 (22.1%)	34 (22.8%)	21 (14.1%)	4 (2.7%)	7 (4.7%)	10 (6.7%)	4 (2.7%)	5 (3.4%)	2 (1.3%)	149 (100%)
1–3 years	30 (15.5%)	33 (17.0%)	22 (11.3%)	25 (12.9%)	15 (7.7%)	17 (8.8%)	18 (9.3%)	17 (8.8%)	10 (5.2%)	7 (3.6%)	194 (100%)
4–6 years	10 (21.3%)	7 (14.9%)	11 (23.4%)	0 (0.0%)	1 (2.1%)	4 (6.4%)	1 (2.1%)	3 (6.4%)	2 (4.3%)	8 (17.0%)	47 (100%)
7–16 years	7 (18.4%)	5 (13.2%)	10 (26.3%)	3 (7.9%)	2 (5.3%)	2 (5.3%)	4 (10.5%)	2 (5.3%)	1 (2.6%)	2 (5.3%)	38 (100%)
Injuries (ICD-10 Code)											
FIB	24 (21.6%)	25 (22.5%)	16 (14.4%)	12 (10.8%)	3 (2.7%)	7 (6.3%)	8 (7.2%)	6 (5.4%)	6 (5.4%)	4 (3.6%)	111 (100%)
Falls	11 (29.7%)	7 (18.9%)	4 (10.8%)	3 (8.1%)	3 (8.1%)	2 (5.4%)	2 (5.4%)	2 (5.4%)	2 (5.4%)	1 (2.7%)	37 (100%)
Burn and scald	3 (10.7%)	5 (17.9%)	4 (14.3%)	4 (14.3%)	2 (7.1%)	5 (17.9%)	2 (7.1%)	2 (7.1%)	1 (3.6%)	0 (0.0%)	28 (100%)
EIMF	4 (8.9%)	9 (20.0%)	6 (13.3%)	5 (11.1%)	3 (6.7%)	3 (6.7%)	5 (11.1%)	7 (15.6%)	1 (2.2%)	2 (4.4%)	45 (100%)
Medical care	21 (15.2%)	23 (16.7%)	37 (26.8%)	17 (12.3%)	6 (4.3%)	9 (6.5%)	9 (6.5%)	6 (4.3%)	6 (4.3%)	4 (2.9%)	138 (100%)
Drug	2 (7.7%)	2 (7.7%)	4 (15.4%)	4 (15.4%)	3 (11.5%)	1 (3.8%)	3 (11.5%)	2 (7.7%)	1 (3.8%)	4 (15.4%)	26 (100%)
Medical device	9 (64.3%)	0 (0.0%)	4 (28.6%)	0 (0.0%)	1 (7.1%)	0 (0.0%)	0 (0.0%)	0 (0.0%)	0 (0.0%)	0 (0.0%)	14 (100%)
Other	2 (6.9%)	7 (5.3%)	2 (5.2%)	4 (13.8%)	1 (3.4%)	3 (10.3%)	4 (13.8%)	1 (3.4%)	1 (3.4%)	4 (13.8%)	29 (100%)
Total	76 (17.8%)	78 (18.2%)	77 (18.0%)	49 (11.4%)	22 (5.1%)	30 (7.0%)	33 (7.7%)	26 (6.1%)	18 (4.2%)	19 (4.4%)	428 (100%)

FIB, fall involving bed; EIMF, exposure to inanimate mechanical forces.

Age stratification revealed that children aged 1–3 years constituted the largest group (*n* = 194, 45.3%), followed by those aged 0–1 year (*n* = 149, 34.8%). Younger children, particularly those under 3 years old, were disproportionately affected, together accounting for over 80% of all cases. The age group 4–6 years represented 11.0% (*n* = 47) of injuries, while children aged 7–16 years accounted for the smallest proportion (*n* = 38, 8.9%).

### 3.2 The trend of unintentional injuries from 2015 to 2024

The demographic composition gender and age distribution and sorts of unintentional injuries are presented in Table 1, with proportions and yearly variations clearly described. There were statistically significant differences in age, and sorts of unintentional injuries among hospitalized children in different years from 2015 to 2024. As illustrated in Table 1, the annual distribution of injuries showed fluctuations, with the highest incidence observed in 2016 (*n* = 78, 18.2%), 2017 (*n* = 77, 18.0%), and 2015 (*n* = 76, 17.8%). A noticeable decreasing trend occurred after 2018, with the lowest number of cases recorded in 2023 (*n* = 18, 4.2%) and 2024 (*n* = 19, 4.4%).

The classification of unintentional injuries based on ICD-10 codes, as well as their temporal trends, is reported in Tables 1, 2, highlighting the changing rankings of major injury categories across years. Analysis of injury sorts based on ICD-10 codes revealed that “Medical care” was the leading cause of injury throughout the study period, accounting for 32.2% (*n* = 138) of all cases, followed by “Fall involving bed (FIB)” (*n* = 111, 25.9%) and “Exposure to inanimate mechanical forces (EIMF)” (*n* = 45, 10.5%). Together, these three categories constituted 68.6% of all unintentional injuries. Annual rankings of the top three injury causes (Table 2) indicated variability across years. For instance, “Medical care” and “FIB” consistently ranked first or second in most years, while “EIMF” and “Burn and scald” also appeared frequently among the top causes. Notably, in 2022, “EIMF” emerged as the leading cause (26.9%), underscoring temporal shifts in injury patterns.

**Table 2:** The variation in the ranking of causes of unintentional injuries among hospitalized children from 2015–2024.

Year	No. 1	No. 2	No. 3	Total (%)
Total	Medical care (138, 32.2%)	FIB (111, 25.9%)	EIMF (45, 10.5%)	68.6
2015	FIB (24, 31.6%)	Medical care (21, 27.6%)	Falls (11, 14.5%)	73.7
2016	FIB (25, 32.1%)	Medical care (23, 29.5%)	EIMF (9, 11.5%)	73.1
2017	Medical care (37, 48.1%)	FIB (16, 20.8%)	EIMF (6, 7.8%)	76.7
2018	Medical care (17, 34.7%)	FIB (12, 24.5%)	EIMF (5, 10.2%)	69.4
2019	Medical care (6, 27.3%)	FIB (3, 13.6%)	EIMF (3, 13.6%)	54.5
2020	Medical care (9, 30.0%)	FIB (7, 23.3%)	Burn and scald (5, 16.7%)	70
2021	Medical care (9, 27.3%)	FIB (8, 24.2%)	EIMF (5, 15.2%)	66.7
2022	EIMF (7, 26.9%)	FIB (6, 23.1%)	Medical care (6, 23.1%)	73.1
2023	FIB (6, 33.3%)	Medical care (6, 33.3%)	Falls (2, 11.1%)	77.7
2024	FIB (4, 21.1%)	Medical care (4, 21.1%)	Drug (4, 21.1%)	63.3

### 3.3 Distribution of unintentional injuries by gender, age, time and place

The distribution of unintentional injuries by gender, age, time, and place is systematically described in Table 3, with chi-square test results included to demonstrate statistical significance. The results showed that the distribution of unintentional injuries in hospitalized children varied significantly by age, time and place.

**Table 3:** The distribution of unintentional injuries by gender, age, time, and place among hospitalized children from 2015 to 2024.

Variables	FIB (*n* = 111)	Falls (*n* = 37)	Burn and scald (*n* = 28)	EIMF (*n* = 45)	Medical care (*n* = 138)	Drug (*n* = 26)	Medical device (*n* = 14)	Other (*n* = 29)	*χ*^2^ value	*p*-value
Gender									5.584	0.558
Male	73 (65.8%)	24 (64.9%)	18 (64.3%)	33 (73.3%)	85 (61.6%)	16 (61.5%)	6 (42.9%)	16 (55.2%)		
Female	38 (34.2%)	13 (35.1%)	10 (35.7%)	12 (26.7%)	53 (38.4%)	10 (38.5%)	8 (57.1%)	13 (44.8%)		
Age									**114.551**	**<0.05**
0–1 year	41 (36.9%)	3 (8.1%)	3 (10.7%)	15 (33.3%)	67 (48.6%)	7 (26.9%)	8 (57.1%)	5 (17.2%)		
1–3 years	64 (57.7%)	28 (75.7%)	22 (78.6%)	23 (51.1%)	34 (24.6%)	7 (26.9%)	2 (14.3%)	14 (48.3%)		
4–6 years	6 (5.4%)	5 (13.5%)	1 (3.6%)	3 (6.7%)	19 (13.8%)	10 (38.5%)	1 (7.1%)	2 (6.9%)		
7–16 years	0 (0.0%)	1 (2.7%)	2 (7.1%)	4 (8.9%)	18 (13.0%)	2 (7.7%)	3 (21.4%)	8 (27.6%)		
Time									**64.045**	**<0.05**
0:00–6:00	22 (19.8%)	0 (0.0%)	0 (0.0%)	2 (4.4%)	28 (20.3%)	1 (3.8%)	2 (14.3%)	0 (0.0%)		
6:00–12:00	22 (19.8%)	7 (18.9%)	2 (7.1%)	13 (28.9%)	46 (33.3%)	7 (26.9%)	6 (42.9%)	13 (44.8%)		
12:00–18:00	24 (21.6%)	15 (40.5%)	10 (35.7%)	19 (42.2%)	35 (25.4%)	9 (34.6%)	4 (28.6%)	7 (24.1%)		
18:00–24:00	43 (38.7%)	15 (40.5%)	16 (57.1%)	11 (24.4%)	29 (21.0%)	9 (34.6%)	2 (14.3%)	9 (31.0%)		
Place									**163.996**	**<0.05**
Ward	110 (99.1%)	20 (54.1%)	23 (82.1%)	37 (82.2%)	135 (97.8%)	24 (92.3%)	13 (92.9%)	23 (79.3%)		
Clinical area	0 (0.0%)	0 (0.0%)	0 (0.0%)	1 (2.2%)	2 (1.4%)	2 (7.7%)	1 (7.1%)	6 (20.7%)		
Out of the ward	0 (0.0%)	11 (29.7%)	3 (10.7%)	7 (15.6%)	1 (0.7%)	0 (0.0%)	0 (0.0%)	0 (0.0%)		
Other	1 (0.9%)	6 (16.2%)	2 (7.1%)	0 (0.0%)	0 (0.0%)	0 (0.0%)	0 (0.0%)	0 (0.0%)		

The bold font represents a statistically significant difference (*p* < 0.05).

Regarding gender, males accounted for a higher proportion of injuries across most categories, including falls involving bed (FIB; 65.8%), exposure to inanimate mechanical forces (EIMF; 73.3%), and medical care-related incidents (61.6%). Females showed a relatively higher rate of injuries related to medical device (57.1%). However, the chi-square test indicated that these gender differences were not statistically significant.

A strong statistically significant association was found between age and injury sort (*χ*^2^ = 114.551, *p* < 0.05). Children aged 1–3 years were most frequently involved in falls (75.7%), burns and scalds (78.6%), and FIB (57.7%). Infants (0–1 year) accounted for nearly half of all medical care-related injuries (48.6%) and over half of injuries involving medical device (57.1%). Older children (7–16 years) were more likely to experience injuries categorized as “other” (27.6%) and showed greater representation in injuries outside typical young child patterns.

Analysis of injury occurrence by time of day revealed significant variation (*χ*^2^ = 64.045, *p* < 0.05). Injuries showed distinct temporal patterns. The evening period (18:00–24:00) accounted for the highest proportion of burns and scalds (57.1%) and a substantial portion of falls (40.5%) and FIB (38.7%). The afternoon period (12:00–18:00) was associated with the highest rate of falls (40.5%) and EIMF (42.2%). The morning period (6:00–12:00) saw the highest proportion of medical device-related injuries (42.9%) and medical care-related incidents (33.3%). The night period (0:00–6:00) was characterized by minimal falls, burns, and scalds, but accounted for 20.3% of medical care-related injuries.

The place of injury occurrence also showed significant variation (*χ*^2^ = 163.996, *p* < 0.05). The vast majority of injuries occurred within the ward, particularly those related to FIB (99.1%) and medical care (97.8%). Falls were likely to occur outside the ward (29.7%), and injuries categorized as “other” showed high proportion within clinical areas (20.7%).

### 3.4 Relationship of injury severity to demographic, time, place, and injury sorts

Then, we further analyzed the relationship between injury severity and demographic, time, place, and injury sorts, with corresponding *χ*^2^ values and *p*-values reported. It is worth noting that no Level I unintentional injuries were identified in this investigation. The association between the severity of unintentional injuries (classified as Level II, III, and IV, with Level II representing the most severe outcomes) and factors including gender, age, time, location, and injury sort is presented in Table 4. Statistical analysis using chi-square tests revealed significant variations in the place of injury occurrence and injury sort, while other factors did not demonstrate significant associations.

**Table 4:** The relationship between unintentional injury levels and gender, age, time, place, and injury sorts among hospitalized children from 2015 to 2024.

Variables	Level II	Level III	Level IV	*χ*^2^ value	*p*-value
Gender				3.629	0.163
Male	0 (0.0%)	1 (50.0%)	270 (63.7%)		
Female	2 (100.0%)	1 (50.0%)	154 (36.3%)		
Age				7.779	0.255
0–1 year	2 (100.0%)	1 (50.0%)	146 (34.4%)		
1–3 years	0 (0.0%)	0 (0.0%)	194 (45.8%)		
4–6 years	0 (0.0%)	1 (50.0%)	46 (10.8%)		
7–16 years	0 (0.0%)	0 (0.0%)	38 (9.0%)		
Time				5.344	0.501
0:00–6:00	1 (50.0%)	0 (0.0%)	54 (12.7%)		
6:00–12:00	1 (50.0%)	1 (50.0%)	114 (26.9%)		
12:00–18:00	0 (0.0%)	1 (50.0%)	122 (28.8%)		
18:00–24:00	0 (0.0%)	0 (0.0%)	134 (31.6%)		
Place				**16.683**	**<0.05**
Ward	1 (50.0%)	2 (100.0%)	382 (90.1%)		
Clinical area	1 (50.0%)	0 (0.0%)	11 (2.6%)		
Out of the ward	0 (0.0%)	0 (0.0%)	22 (5.2%)		
Other	0 (0.0%)	0 (0.0%)	9 (2.1%)		
Injuries (ICD-10 code)				**25.926**	**< 0.05**
FIB	0 (0.0%)	0 (0.0%)	111 (26.2%)		
Falls	0 (0.0%)	0 (0.0%)	37 (8.7%)		
Burn and scald	0 (0.0%)	0 (0.0%)	28 (6.6%)		
EIMF	0 (0.0%)	1 (50.0%)	44 (10.4%)		
Medical care	1 (50.0%)	0 (0.0%)	137 (32.3%)		
Drug	0 (0.0%)	1 (50.0%)	25 (5.9%)		
Medical device	1 (50.0%)	0 (0.0%)	13 (3.1%)		
Other	0 (0.0%)	0 (0.0%)	29 (6.8%)		

The bold font represents a statistically significant difference (*p* < 0.05).

No statistically significant differences in injury severity were observed by gender (*χ*^2^ = 3.629, *p* = 0.163), though all Level II injuries occurred in females. Similarly, age distribution did not significantly affect injury severity level (*χ*^2^ = 7.779, *p* = 0.255). Infants (0–1 year) accounted for all Level II injuries and half of Level III injuries. The time of injury also showed no significant correlation with severity (*χ*^2^ = 5.344, *p* = 0.501).

In contrast, the place of injury occurrence was significantly associated with severity (*χ*^2^ = 16.683, *p* < 0.05). Level II injuries occurred equally in wards and clinical areas (50.0% each), while Level III injuries exclusively occurred in wards (100.0%). The vast majority (90.1%) of Level IV injuries, the least severe, also occurred in wards.

A strong significant relationship was identified between injury sort and severity (*χ*^2^ = 25.926, *p* < 0.05). Level II injuries were associated with “Medical care” (50.0%) and “Medical device” (50.0%). Level III injuries occurred with “EIMF” (50.0%) and “Drug”-related events (50.0%). Level IV injuries, which constituted the majority of cases, were distributed across all injury categories, most notably “FIB” (26.2%), “Medical care” (32.3%), and “EIMF” (10.4%).

These results indicate that while demographic and temporal factors were not major determinants of injury severity in this cohort, the location and specific type of injury were significantly associated with the severity of outcomes. This underscores the importance of focusing prevention strategies on particular environments and mechanisms of injury to reduce the incidence of more severe harm.

## 4 Discussion

This study systematically analyzed 428 cases of unintentional injuries among hospitalized children, identifying key epidemiological patterns. Contrary to previous reports emphasizing falls (8.6%) as the primary injury mechanism (18, 19), falling from beds (25.9%) emerged as the predominant cause in our cohort. Unintentional injuries remain the leading cause of mortality among children and adolescents aged 0–19 years, with falls, mechanical force exposure, other unintentional injuries, foreign body incidents, and animal contact representing the top five fatal mechanisms in 2019 (20). Consistent with global patterns, male patients sustained approximately twice as many injuries as females, and children aged 0–4 exhibited the highest rates of both fatal and disabling injuries.

According to the National Electronic Injury Monitoring System of the United States from 2007 to 2021, falling, jumping and falling into bed are the main injury mechanisms for children under 5 years of age (21). However, international variations exist: a survey in Japan among 400 family institutions reported only 36 falls (1.7%) among 1937 adverse events within 3 months (22), while a Swedish study found falls accounted for 18.8% of all adverse events (23). A national study in Canada reported injury falls (17.2%) as the most common adverse events (24). These discrepancies may be related to differences in disease composition, medical operation habits, and healthcare infrastructure across regions.

The gender distribution in our study showed 271 cases (63.32%) in male children and 157 cases (36.68%) in females, consistent with most related studies domestically and internationally (25). This pattern may be attributed to boys’ more active nature and greater exploratory behavior, increasing their vulnerability to unintentional injuries during hospitalization. Regarding age distribution, children aged 1–3 years experienced the highest incidence (40.4%), followed by infants aged 0–1 year (32.0%). This finding aligns with a Japanese study reporting that adverse events were common in pediatric inpatients with an incidence of 76 per 1,000 patient days, and 37% of pediatric inpatients had at least 1 adverse event during hospitalization (26). Children at this developmental stage exhibit strong curiosity but insufficient danger recognition and self-protection capabilities. Additionally, hospitalized children, especially young children, often have poor physical balance and coordination, and the inpatient environment may cause anxiety and fear, leading to abnormal behaviors and increased accident risk (27).

The hospital environment was identified as a significant factor in unintentional injuries. Wards were high-incidence locations, where floor conditions and facility layout increased risks. Slippery floors, broken or unfixed bed rails, and suboptimal furniture arrangement contributed to fall incidents. Compared with hospitals with advanced management, medical centers in this region may have deficiencies in environmental maintenance, including untimely stain cleaning and inadequate equipment inspection. Although burns accounted for a relatively low proportion, their impact should not be overlooked, potentially resulting from poorly managed hot water supply systems and insufficient awareness of hot water dangers among children and parents.

Temporal patterns revealed that 18:01–24:00 (32.0%) and 12:01–18:00 (28.7%) were high-incidence periods, collectively accounting for over 60% of injuries. This distribution challenges traditional assumptions about nighttime risk and may be explained by decreased staffing during shift changes (17:30–18:15), parental distraction during meal preparations, and increased child activity during evening hours due to hunger or irritability. Environmental factors such as insufficient illumination, frequent personnel movement, and parental fatigue further compromised supervision quality.

Based on these findings, we recommend multifaceted prevention strategies. Evidence exists for interventions to prevent infection, falls, delirium, adverse drug events, cardiopulmonary arrest, and death (28). Hospitals should implement regular safety inspections, adjust bed heights to meet safety standards, install protective pads on bed rails, maintain dry and clean floors, and place non-slip mats in slippery areas. Staffing adjustments should ensure adequate nursing coverage during high-incidence periods, with increased rounding frequency from 17:30 to 18:15 to promptly identify and stop dangerous behaviors. Parental education is equally crucial. Hospitals should regularly organize safety education through lectures, videos, and brochures to enhance parental awareness of common unintentional injuries and preventive measures (29). Parents should be informed about proper use of hot water equipment, dangerous goods safekeeping, and enhanced child supervision. Encouraging active parental participation in child safety management and close collaboration with medical staff is essential (30).

Although most injuries in this study were mild, a small proportion of moderate injuries occurred that may seriously affect children’s physical and mental health. Prevention efforts should therefore focus not only on reducing incidence but also on minimizing severe injury events (31). Hospitals should improve unintentional injury reporting systems to ensure timely and accurate documentation, regularly analyze reported events to identify patterns and causes, and evaluate prevention measure effectiveness. These data should be feedback to all healthcare staff and relevant departments to enhance organization-wide attention to child safety and promote interdepartmental cooperation (25).

Furthermore, the measurement of adverse events must be part of the learning system of health-care organizations and linked to evidence-based interventions and evaluation of these interventions as part of continuous improvement efforts, as measurement alone cannot create safe care (32). Successful examples include education and consistent risk stratification using bed/chair alarms at a community hospital in the southeastern United States that reduced falls from 8.67 to 5.07 per 1,000 patient days (33), and the use of an out-of-bed alarm at an emergency care hospital in Singapore that was associated with reduced fall incidence (34).

However, this study has several limitations. First, the retrospective and single-center nature of our research inherently restricts the generalizability of our results. The data collected may reflect the specific practices, patient demographics, and institutional characteristics of our hospital, which may not be fully representative of other settings. Furthermore, we recognize the potential for significant underreporting, particularly concerning minor injuries. Such incidents might not have been documented in the medical records if they did not result in substantial clinical consequences or require specific medical intervention, leading to a possible underestimation of the true incidence rate. Our dataset lacked certain critical details that could provide deeper contextual insights. These include specific disease diagnoses for all patients, the status of caregivers (e.g., levels of fatigue or inattention at the time of the incident), and precise details about ward facilities and environmental factors. The absence of these variables limits our ability to perform a more granular analysis of the root causes and contributing factors behind the reported events. Despite these limitations, our findings offer valuable preliminary insight. Future research should adopt prospective, multi-center designs to validate these findings and develop targeted interventions for different healthcare settings. Implementation studies evaluating the effectiveness of specific preventive measures are essential for translating evidence into practice.

## 5 Conclusion

In conclusion, this study elucidates the demographic, temporal, and environmental determinants of unintentional injuries among hospitalized children in western China. The findings provide valuable insights for developing targeted prevention strategies and contribute to the ongoing efforts to enhance pediatric inpatient safety globally.

## Data Availability

The original contributions presented in the study are included in the article/supplementary material, further inquiries can be directed to the corresponding authors.
